# Knowledge and misconceptions of parents of children with attention-deficit hyperactivity disorder at a hospital in South Africa

**DOI:** 10.4102/safp.v62i1.5124

**Published:** 2020-09-03

**Authors:** Neelkant R. Rajcumar, Saeeda Paruk

**Affiliations:** 1 Discipline of Psychiatry, University of KwaZulu-Natal, Durban, South Africa

**Keywords:** misconceptions, cultural factors, KwaZulu-Natal, perception, child psychiatry, treatment, African mental healthcare setting

## Abstract

**Background:**

Parents’ knowledge and misconception about attention-deficit hyperactivity disorder (ADHD) influences their children’s access to care, its management and outcome. The study aimed to investigate parents’ knowledge and perceptions of ADHD.

**Methods:**

The cross-sectional survey of 79 parents of children (aged 5–17 years) with ADHD at a specialist child psychiatry clinic in KwaZulu-Natal Province, South Africa, consisted of a socio-demographic and clinical questionnaire, and the Knowledge of Attention Disorders Scale questionnaire, was carried out.

**Results:**

Twenty-six (32.9%) parents consulted a traditional healer, of whom 84.6% did so before consulting a medical doctor, with 61.5% reporting that the healer suggested psychiatric referral. Most parents had some knowledge of their child’s ADHD diagnosis but held various misconceptions about its treatment and associated factors: 92.4% believed that reducing sugar or food additives were effective to reduce symptoms; 78.5% that treatments focussing on punishment reduced the symptoms; 67.1% that prolonged use of stimulant medications leads to increased addiction (i.e. drug, alcohol) in adulthood.

**Conclusion:**

Many parents had misconceptions about ADHD’s causes and treatment, some having consulted traditional healers, indicating the need to increase awareness among health practitioners to ensure timeous treatment access. A parent focussed psycho-education programme is required that provides information about causes, symptoms, treatment and prognosis.

## Introduction

Parental knowledge, attitude or perception about psychiatric conditions is essential for young children, as these are the people who rely to access care, ensure treatment adherence and provide support to improve the outcomes.^1^ According to the Health Belief Model,^2^ knowledge of mental conditions and their management determines treatment-seeking behaviour, this being key to improving the outcomes for children.^3^ Attention-deficit hyperactivity disorder (ADHD) is one of the most common childhood-onset psychiatric disorders and has a stable worldwide prevalence of 5% – 7%,^4,5,6^ including in Africa^5^ and South Africa.^6^

Attention-deficit hyperactivity disorder accounts for a significant number of child referrals to psychiatric and paediatric mental health services. Although the incidence of ADHD is comparable in both white and African Americans in the United States (US), fewer children of the latter were diagnosed and treated, which may be because of the complex barriers extending from the home to the health care system.^7^ Factors such as education and awareness programmes, cultural and public perceptions, physician characteristics, parent knowledge and attitude or perception and various environmental exposures may influence access to and the quality of care that children with ADHD receive.^8^ Poor or inadequate commitment by parents to ADHD management will result in inadequate treatment adherence to pharmacological and non-pharmacological therapies, or even its discontinuation.^9^ Parental commitment to treatment may, in turn, be influenced by several factors, including knowledge of and attitude to ADHD, socio-economic status, stress, coping mechanisms and cultural factors.^4,10^

Although it has been scientifically accepted that ADHD is a childhood-onset neurobiological disorder^11^ and that medication is the mainstay of treatment,^12^ public controversy about it remains prevalent, being fuelled by perceptions from society, including parents, teachers and even health professionals.^13^ There are also many misconceptions relating to its causes, symptoms, treatment and course.^10^ Misconceptions commonly relate to the sources of information, for example, television and social media, parenting practices and the side effect profiles of psychotropic medications, particularly the stimulant methylphenidate. Based on these perceptions, parents tend to balance the advantages and disadvantages of using ADHD medications, with some opting for less evidence-based treatment.^1,14,15^

In an Australian community study of parents and adolescents, while many participants were knowledgeable about ADHD, there were gaps in their knowledge about its genetic aetiology and course of illness, with many caregivers expressing concerns about over-medication.^15^ A community study in the US on adolescents at high risk of developing ADHD because of family history reported that, although many of their parents, teachers and health care professionals were familiar with the diagnosis, there were several misconceptions relating to its treatment and the potential addictive effect of medication.^16^ This has been further supported by a review of the literature on surveys among parents, teachers and professional regarding ADHD.^13^

A study in Iran in 2007 of parents of children with ADHD found that their knowledge of the condition was relatively poor, particularly relating to its biological and genetic causation and their limited awareness led to delays in accessing care.^17^ Although a South African study assessed primary school teachers’ knowledge and misperceptions of ADHD in the Western Cape province,^18^ there appears to be very limited data from Africa on the subject.

Parental knowledge of ADHD may be influenced by multiple psycho-socio-cultural factors, including educational level, race, culture, stigma, access to the Internet and mental health services.^19^ Bussing et al. suggested that cultural variations may affect ADHD knowledge in the US,^1^ although this was not borne out in a later study.^16^ In a more recent (2017) study in Iran, parental knowledge in a clinical sample correlated significantly with educational level.^10^ In addition, parental knowledge, attitudes and awareness may also be influenced by the information sources, with patients and carers traditionally relying on the physician for psycho-education, although there is an increasing reliance on the Internet and other media.^13^ In the more recent Iranian study by Dodangi and colleagues, 52% of parents of affected children reported that the television was the most common source of information.^10^

The literature therefore notes that several misconceptions about ADHD persist, specifically related to diagnosis and treatment, which may be influenced by various factors that, in turn, could affect adherence and outcome. Against this backdrop of ADHD being one of most common neuro-developmental disorders in children, and because of the reliance on parental knowledge and attitudes to its treatment to ensure an optimal outcome, it is important to investigate this aspect in the South African setting, where there are limited mental health resources.

This study therefore aimed to describe the knowledge and misconceptions towards ADHD among parents of children receiving care at a public sector outpatient psychiatric hospital in KwaZulu-Natal province, South Africa. We also aimed to explore any associations between parents’ socio-demographic characteristics and the Knowledge of Attention Deficit Disorder Scale (KADDS) sub-scale scores or misconceptions.

## Method

### Participants

This cross-sectional, descriptive study involved a questionnaire interview with 79 adults (18 years and older) biological parents of children (aged 5–17 years) with an established clinical diagnosis of ADHD, as per the Diagnostic and Statistical Manual (DSM 5) criteria^20^ who were referred to study researcher. Inclusion criteria were English-speaking parents of children with ADHD at a specialist child psychiatry outpatient service and had been under treatment for a minimum of 6 months. The diagnosis of ADHD was based on the clinical assessment by using DSM 5 criteria by senior psychiatry registrars under the supervision of or by a child psychiatrist. Carers who were not biological parents were not included in this study.

All parents were invited to participate in the study, with survey being completed on their clinical visit. All surveys were completed by the principal investigator (N.R.) after written informed consent was obtained, with data being collected from May 2017 to March 2018.

#### Instruments

Two instruments were used to obtain data: a parent socio-demographic questionnaire and the KADDS.

**Socio-demographic and clinical questionnaire:** A socio-demographic and clinical questionnaire was used to obtain child and parent variables and consisted of three sections. Section 1 obtained the parent’s demographic details, such as age, gender, occupation, educational level, household income and the number of children. They were also asked if they had consulted a faith healer and whether they had suggested psychiatric treatment. Section 2 obtained the child’s socio-demographic (age, gender, educational level) and clinical characteristics from the parents, which were confirmed by chart review for variables, such as diagnosis, duration of illness and treatment. Section 3 explored the sources of parents’ knowledge and misconceptions about ADHD and was based on a review of the literature. Data on their experience of stigmatisation, support systems, ADHD causes, sources of information and perception of the health care received were collated. Stigma was defined for parents as a mark of shame, disgrace or disapproval that results in an individual being rejected, discriminated against and excluded from main society.

**Knowledge of Attention Deficit Disorder Scale:** The KADDS is a 36-item rating-scale questionnaire^21^ that uses a true, false or do not know format and enables a comparison between the parent’s knowledge and their attitudes (perceptions), that is, their misconceptions. The KADDS was developed to measure knowledge and misconceptions in three sub-scales: symptom or diagnosis (9 items), treatment (12 items) and associated features (i.e. general knowledge about the nature, causes and outcome) (15 items) among parents, teachers and mental health professionals, being used in this study for the former only. Knowledge was measured by correct scores and misconceptions by incorrect responses.

The questionnaire does not make provision for a total score, with each item being assessed individually and the sub-scale scores being compared. The items in the KADDS scale have statistical reliability and validity, with research having been conducted on the internal consistency of its total score, based on the original 36 items scale (three items are still for further testing), revealing a high internal consistency, ranging from 0.81 to 0.86.^18^ Internal consistency was found in the present study, with the Cronbach’s alpha for the total score being 0.551, possibly because of poorer proficiency in English as English may have a second language for some participants. The KADDS scale was used previously in the Western Cape Province, South Africa, in a study that assessed primary school teachers’ knowledge and misconceptions of ADHD.^18^ The tools were piloted on 10 parents prior to data collection to ensure that all questions were comprehensive. It provided insight on how best to phrase the survey questions and to ensure that they were all understood.

### Statistical analysis

The quantitative data from the socio-demographic questionnaire were entered and analysed by using the Statistical Package for Social Sciences version 26 software by using descriptive statistics. The variables from the KADDS questionnaire were also descriptively analysed by using the number and per cent for the categorical variables, with their frequency distribution being examined, and the means with standard deviation, medians and inter-quartiles ranges being used, as appropriate. The three sub-groups of the KADDS (knowledge and misconceptions about diagnosis, treatment and associated factors) were compared by conducting chi-square tests on *t*-tests or Wilcoxon rank sum test, with a *p* < 0.05 being considered statistically significant. The data are presented in three sections: (1) correct knowledge, (2) incorrect knowledge (misconceptions) and (3) associations between misconceptions with parent characteristics.

### Ethical consideration

The study was approved by the University of KwaZulu-Natal’s Biomedical Research Ethics Committee (Ref BE036/17). Permission was also obtained from the Department of Health and written informed consent from the parents.

## Results

Of the 82 parents who were invited to participate, 79 agreed and 3 refused. The results were: (1) the parents’ socio-demographic and health-seeking behaviour, (2) children’s clinical characteristics, (3) the source of the parents’ knowledge, being followed by (4) the KADDS sub-scale scores (knowledge and misconceptions) and possible associations between the parent factors.

### Parents’ socio-demographic characteristics and health-seeking behaviour

The majority of parents were 41–50 years old (49.4%), female (*n* = 57, 72.2%), married (59.5%) and black (49.4%); did not have a tertiary education (*n* = 66, 83.5%); and had two children (45.6%). Seven (8.9%) of the parents had two or more children with ADHD, whereas the majority (*n* = 71, 91.1%) had only one affected child (Table 1). The sociodemographic data of the parents are presented in Table 1.

**TABLE 1:** Parents’ socio-demographic characteristics (*n* = 79).

Variable	*n*	%
**Age (years)**		
18–30	8	10.1
31–40	23	29.1
41–50	39	49.4
51–60	9	11.4
**Gender**		
Male	22	27.8
Female	57	72.2
**Marital status**		
Single	15	19.0
Married	47	59.5
Divorced or widowed	17	21.5
**Race**		
Black	39	49.4
White	6	7.6
Indian	12	15.2
Mixed race	22	27.8
**Tertiary education**		
Yes	13	16.5
No	66	83.5
**Highest educational level†**		
Grades 1–6	3	3.7
Grades 7–9	18	22.7
Grades 10–12	58	73.4
**Number of children**		
1	27	34.2
2	36	45.6
3	12	15.1
4 or more	4	5.1
**Number of children with ADHD**		
1	72	91.1
2 or more	7	8.9
**Employment**		
Employed	36	45.6
Unemployed	43	54.4
**Monthly income (South African Rand)**		
1000–2500	19	24.1
2501–5000	20	25.3
5001–9999	25	31.6
> 10 000	15	19.0

ADHD, attention-deficit hyperactivity disorder; SD, standard deviation.

† , Mean and SD = Grade 10 and SD = 1.87.

### Consultation with a faith healer

Table 2 shows the summary of the data on those parents who consulted a faith healer about their child’s condition, with 26 (32.9%) parents indicating that they had done so. Twenty-two (84.6%) of the 26 parents had done so before seeking medical treatment, eight (36.4%) having found it useful and 16 (76%) noting that they had been advised to seek psychiatric treatment at a health facility.

**TABLE 2:** Consultation with a faith healer (*n* = 26 of the 79 parents).

Variable	Characteristic	No.	%
Consulted a faith healer	Yes	26	32.9
	No	53	67.1
If yes,	Before medical treatment	22	84.6
	After medical treatment	3	11.5
	Not sure	1	3.8
Faith healer being useful	Yes	8	36.4
	No	7	31.8
	Not sure	7	31.8
Faith healer suggesting psychiatric treatment	Yes	16	76.2
	No	5	23.8
	Did not know	5	23.8
Faith healer’s consultation charge (South African Rand)	Free	5	21.7
	100	1	4.3
	150	5	21.7
	200	8	34.8
	250	1	4.3
	300	2	8.7
	450	1	4.3
	Did not remember	3	13

### Children’s demographic and clinical characteristics

Table 3 indicates that most of the 90 children were male (*n* = 74, 82.2%). In terms of age, 57.7% were 10 years and older, 67.7% (*n* = 61) attended mainstream schools, and although 87.7% had been diagnosed at least 2 years previously, 95.5% had been on treatment for more than a year.

**TABLE 3:** Demographic and clinical characteristics of children with attention-deficit hyperactivity disorder (*n* = 90).

Variable	*n*	%
**Gender**		
Male	74	82.2
Female	16	17.8
**Child age (years)**		
< 5	0	0.0
5–6	18	20.0
7–9	20	22.2
10–12	28	31.1
> 12	24	26.6
**Education**		
No school	2	2.2
Remedial school	27	30.0
Mainstream school	61	67.7
**Duration of ADHD illness**		
≤ 2 years	11	12.2
> 2 years	79	87.7
**Duration of ADHD treatment**		
≤ 1 year	4	4.4
> 1 year	86	95.5

Note: The mean grade for children’s level of education was Grade 4 with a standard deviation of 2.10.

ADHD, attention-deficit hyperactivity disorder.

### Source of attention-deficit hyperactivity disorder information

All the parents (*N* = 79, 100%) reported being informed of the diagnosis of ADHD by a medical doctor, with the majority being satisfied about obtaining information on the diagnosis (*N* = 77, 97.4%) and treatment (*N* = 76, 96.2%), but data on type and quality of information received were not collated. However, the majority reported experiencing stigma (*N* = 60, 75.9%) relating to their child’s illness. Although doctors (*n* = 79, 100%) and clinic staff (*n* = 79, 100%) were the most common providers of information, there were other sources (*n* = 79, 100%), such as teachers (*n* = 71, 89.8%), the media (*n* = 70. 88, 6%) (newspaper and television), the Internet (*n* = 45, 56.9%) and other parents (*n* = 36, 45.5%). Fifty-seven (72.1%) parents mainly attributed the cause of ADHD to a chemical imbalance in the brain, 21 (26.5%) to genetics, 27 (34.1%) to sugar use and 21 (26.6%) to being a spoilt child.

### KADDS: Parent’s knowledge and misconception scores

The participants’ KADDS scores (correct responses) were assessed for the three sub-scale scores and individual items and are summarised in Table 4.

**TABLE 4:** Parents’ mean correct scores per sub-scale of Knowledge of Attention Deficit Disorder Scale (*n* = 79).

KADDS sub-scales	Items per sub-scale or maximum score	Parent mean correct score	Standard deviation
Symptom or diagnosis	9	6.43	1.599
Treatment	12	6.94	2.102
Associated features	15	6.10	2.421

KADDS, Knowledge of Attention Deficit Disorder Scale.

The most common correct knowledge and incorrect responses (misconceptions) are indicated with their respective question (Q) number and statement from the KADDS.

- **Most common correct knowledge responses:**
  ■ 78 (98.7%) – ADHD children are frequently distracted by extraneous stimuli (Q 3).
  ■ 75 (94.9%) – Side effects of stimulant drugs include insomnia and appetite reduction (Q 15).
  ■ 73 (92.4%) – In order to be diagnosed as ADHD, a child must exhibit relevant symptoms in two or more settings (e.g. home, school) (Q 21).
  ■ 65 (82.3%) – Children with ADHD are more distinguishable from normal children in a classroom setting than in a free play situation (Q 31).
  ■ 53 (67.1%) – Stimulant drugs are the most common type of drug used to treat children with ADHD (Q 25).
- **Most common incorrect responses on KADDS (misconceptions):**
  ■ 70 (88.6%) – When an ADHD child’s treatment is terminated, it is rare for the symptoms to return (Q 12).
  ■ 63 (79.7%) – Most ADHD children will ‘outgrow’ their symptoms by the onset of puberty and function normally in adulthood (Q 19).
  ■ 73 (92.4%) – Reducing sugar or food additives effectively decreases ADHD symptoms (Q 23).
  ■ 60 (75.9%) – Children with ADHD generally experience more problems in novel situations than in familiar situations (Q 27).
  ■ 62 (78.5%) – Treatment that focusses mainly on punishment is the most effective for reducing ADHD symptoms (Q 36).
  ■ 53 (67.1%) – Prolonged use of stimulant medications leads to increased addiction (i.e. drug, alcohol) in adulthood (Q 37).
  ■ 70 (88.6%) – If a child responds to stimulant medications (e.g. Ritalin), they probably have ADHD (Q 38).
- **Association between KADDS sub-scale scores with parent characteristics:**

The small sample size must be noted as a limiting factor.

In addition, the analysis of the parent’s socio-demographic factors and KADDS sub-scale scores is summarised in Table 5.

**TABLE 5:** Association of Knowledge of Attention Deficit Disorder Scale sub-scale score (knowledge) with parental factors.

Variable	Statistical test	Sub-scale 1 associated features	Sub-scale 2 symptom diagnosis	Sub-scale 3 treatment
Parent’s gender	Pearson chi-square	20.097	10.025	16.289
	Sig. (2-tailed)	0.389	0.348	0.363
	*N*	79	79	79
Parent’s marital status	Pearson chi-square	83.285	26.600	53.615
	Sig. (2-tailed)	0.013	0.486	0.177
	*N*	79	79	79
Parent’s tertiary education	Pearson chi-square	23.444	15.446	29.157
	Sig. (2-tailed)	0.218	0.079	0.015
	*N*	79	79	79

Sig., significance.

## Discussion

The aim of this study was to determine the knowledge and misconceptions of ADHD among parents of children receiving treatment for ADHD at an urban public sector psychiatric unit in KwaZulu-Natal, and to establish any associations with their socio-demographic characteristics. The key findings of this study were that parents of children with ADHD all had knowledge of the diagnosis of ADHD, and despite majority believing that they had received information on diagnosis and treatment from the clinic, there was gaps in knowledge on symptoms, treatment and associated features as evidenced by the mean scores for the three sub-scales on KADDS. Misconceptions about punishment as a treatment modality for ADHD, sugar intake associated with symptoms, stimulants causing addiction and ‘outgrowing’ ADHD were most common. In addition, almost a third of parents consulted a traditional healer with support clinical staff being the main source of knowledge on ADHD.

### Consultation with a faith healer

As per the World Health Organization, only 10% of people with neuropsychiatric conditions are estimated to have access to mental health services.^22^ In South Africa, there is a glaring lack of child mental health services, with faith healers playing an important role in trying to bridge that gap, as they are generally accessible. Providing mental health education for faith healers may facilitate appropriate and timeous referral faith healers maybe the first point of contact for parents when seeking an understanding of unusual behaviour.^23^ In this study, 32.9% of parents seeking medical care at an urban facility consulted a traditional healer, further emphasising the critical role they play in the local socio-cultural context and the need to integrate them in a holistic health service. No studies on the extent of traditional health-seeking behaviour for children with ADHD were found.

### Parents’ source of attention-deficit hyperactivity disorder information

All parents (100%) in this study claimed to have received information from the medical staff of the clinic, the other most common sources being schoolteachers (89.8%), the media (88.6%) and Internet (56.9%). Other studies reported the media (radio and television)^10,24^ as being common sources, which suggests that access to suitably trained medical staff may vary between countries. The most common source of information in other studies is the media (radio and television),^10,24^ which is in contrast to the findings of this study where all parents claimed to have received information related to ADHD from the treating medical staff of the clinic. Media is the third most common cause of information in this study. This may represent a form of subjective bias reporting from parents or suggests that parents in this context have less access to media for health education. This suggests that health care services need to ensure that appropriate psychoeducation and support around ADHD is provided as they appear to be the main source of information. This is critical in primary health care settings where parents may have even greater challenges accessing reliable knowledge sources.

### Knowledge of attention-deficit hyperactivity disorder and associated factors

Although all parents were aware of the ADHD diagnosis, the mean scores for the three sub-scales of 6.1 (symptom or diagnosis), 6.4 (treatment) and 6.9 (associated features) (maximum score 15, 9 and 12) suggest that there were still gaps in their knowledge.

These scores are consistent with the literature, which demonstrated that parents have knowledge but still harbour misconceptions relating to ADHD.^13,15,16^ The current study found no association between educational level with improved knowledge on any KADDS sub-scales. This is contrary to the literature that higher education is a positive factor in health-seeking behaviour.^25^ Traditionally studies suggest that while the parents recognised that there could be a behavioural problem with their children, their own educational levels may affect their health-seeking behaviour with relevant health authorities. Parents of lower educational and income levels appear to be less knowledgeable about appropriate treatment behaviour for their children in other international studies;^1,26,27^ however, possibly, access to a specialist service with more individualised care may have modified this finding. The lack of positive association between education and better knowledge of symptom and treatment sub-scales suggests the need to in addition further explore this and other parental variables in a larger study that is possibly better placed at community-level services as access to care may also be measured in those contexts.

### Misconceptions on Knowledge of Attention Deficit Disorder Scale towards attention-deficit hyperactivity disorder

In line with other studies, this study showed some common parental misconceptions that are present in research from developed and developing countries.^10,16^ The misconceptions regarding the sugar intake-associated symptoms of ADHD (most common misconception in this study), use of stimulant leading to addiction, reliance on punishment to bring behavioural change and outgrowing ADHD were common in this and other studies.^28,29,30,31,32,33^ An earlier American study suggested that black Americans strongly accepted the ADHD ‘sugar’ aetiology.^30^ In the study the authors proposed that this may be because of factors such as different cultural thresholds and perception of bad behaviour, beliefs that ADHD is not a medical condition, discrimination, lack of trust of health professionals and economic constraints based on socio-cultural differences.^31,32^ Perold and colleagues reported similar findings relating to ADHD and the sugar myth amongst teachers in South Africa, who believed that sugar was the cause and the children would outgrow the symptoms.^18^ This suggests that as health care workers, we need to provide more information on the role of diet, particularly sugar and ADHD symptoms. There is also a need for more and better qualitative studies to understand the influences that drive these misconceptions.

Another common misconception was related to the addiction potential of stimulants, as this may be associated with poorer medication adherence if the parents have concerns about the treatment options. In this study, 67% of parents believed that a prolonged use of stimulant medications leads to increased risk of substance addiction (i.e. drug, alcohol) in adulthood, and these findings being consistent with the literature.^30,34^ This is an important misconception that should be more actively focussed in psychoeducation as it has treatment and prognostic implications. Parents need to be aware that untreated ADHD is a risk factor for substance use.^35^ This study did not further explore the associations with regard to misconceptions and parental factors as the study sample size limits credibility of findings.

The findings of this study have important implications in planning various interventions about ADHD education, as parents are the most significant role players who influence child health outcomes. By providing accurate knowledge, relating to misconceptions, myths and misperceptions will fade and potentially alter the health-seeking behaviour of parents with children of ADHD. Ideally, paediatric mental health care should be integrated and available at community-based services to improve access and allow early referral of children requiring more complex care to specialist services and hence better utilising scarce mental health resources. This will enable parents to have better problem recognition, selection and utilisation of health resources.^3^ The psycho-education content on ADHD therefore needs to be reviewed to address common myths held by parents. We also need to consider if the gaps are because of insufficient staff training in communication skills and ADHD knowledge. We also need to provide information – including tailored information, avoiding use of jargon and inviting questions for clarification and providing psycho-education in the first language such as isiZulu for parents so that it bridges their gaps in knowledge.

## Limitations

The study had several limitations such as the small sample size, which limit generalisability, and the study setting being in an urban area and at a specialist hospital, which may prevent the results being generalisable to other settings. The inclusion of English-speaking parents only may have biased the sample, as many people attending public sector facilities speak isiZulu as their first language, their knowledge and misconceptions were not captured and they may have been more vulnerable to poorer psycho-education as some information may be lost in translation. The convenience sampling and the participants’ profile are not representative of the national South African demography that may further limit the generalisation of the findings. Although the KADDS has been used in the South African setting, the lack of a validated multilingual tool to assess ADHD knowledge and misconceptions in this setting is another limitation. However, the study does provide insight into parent’s knowledge and perceptions of ADHD in an African mental health care setting.

## Recommendations

There is a need for more focussed psychosocial interventions to address the gaps in knowledge and misconceptions held by parents of children with ADHD. A community-based participatory research study would be useful to determine needs and more appropriate, sustainable interventions.

## Conclusion

The study findings reveal substantial knowledge gaps among parents of children with ADHD regarding treatments and associated features of ADHD and the need to consider more appropriate interventions to address these better in clinical settings including involving appropriate staff and intervention planning.
